# Association between waist circumference and lung function in American middle-aged and older adults: findings from NHANES 2007–2012

**DOI:** 10.1186/s41043-024-00592-6

**Published:** 2024-06-26

**Authors:** Zichen Xu, Lingdan Zhuang, Lei Li, Luqing Jiang, Jianjun Huang, Daoqin Liu, Qiwen Wu

**Affiliations:** 1 Department of Laboratory Medicine, The First Affiliated Hospital of Wannan Medical College, 2 Zheshan West Road, Wuhu, Anhui Province China; 2 Department of Kidney Medicine, The First Affiliated Hospital of Wannan Medical College, 2 Zheshan West Road, Wuhu, Anhui Province China

**Keywords:** Abdominal obesity, Waist circumference, Lung function, National health and Nutrition examination survey

## Abstract

**Purpose:**

There is a major epidemic of obesity, and many obese patients suffer from respiratory symptoms and disease. However, limited research explores the associations between abdominal obesity and lung function indices, yielding mixed results. This study aims to analyze the association between waist circumference (WC), an easily measurable marker of abdominal obesity, and lung function parameters in middle-aged and older adults using the National Health and Nutrition Examination Survey (NHANES).

**Methods:**

This study utilized data obtained from the National Health and Nutrition Examination Survey (NHANES) spanning 2007 to 2012, with a total sample size of 6089 individuals. A weighted multiple regression analysis was conducted to assess the relationship between WC and three pulmonary function parameters. Additionally, a weighted generalized additive model and smooth curve fitting were applied to capture any potential nonlinear relationship within this association.

**Results:**

After considering all confounding variables, it was observed that for each unit increase in WC, in males, Forced Vital Capacity (FVC) increased by 23.687 ml, Forced Expiratory Volume in one second (FEV1) increased by 12.029 ml, and the FEV1/FVC ratio decreased by 0.140%. In females, an increase in waist circumference by one unit resulted in an FVC increase of 6.583 ml and an FEV1 increase of 4.453 ml. In the overall population, each unit increase in waist circumference led to a FVC increase of 12.014 ml, an FEV1 increase of 6.557 ml, and a decrease in the FEV1/FVC ratio by 0.076%. By constructing a smooth curve, we identified a positive correlation between waist circumference and FVC and FEV1. Conversely, there was a negative correlation between waist circumference and the FEV1/FVC ratio.

**Conclusions:**

Our findings indicate that in the fully adjusted model, waist circumference, independent of BMI, positively correlates with FVC and FEV1 while exhibiting a negative correlation with FEV1/FVC among middle-aged and older adults in the United States. These results underscore the importance of considering abdominal obesity as a potential factor influencing lung function in American middle-aged and older adults.

**Supplementary Information:**

The online version contains supplementary material available at 10.1186/s41043-024-00592-6.

## Introduction

Obesity has emerged as a significant global public health challenge. Obesity has markedly increased in over 70 countries since 1980 and continues to rise in most others [1, 2]. In 2015, the global population of individuals classified as obese surpassed one-third [3], and this number is projected to reach a staggering 1.12 billion by 2030 [4]. Obesity constitutes a substantial risk factor for numerous ailments, including metabolic disorders, cardiovascular and cerebrovascular diseases, dyslipidemia, asthma, chronic obstructive pulmonary disease (COPD), and cancer [5–8]. Obesity is commonly categorized into two types: abdominal obesity, assessed by waist circumference, and general obesity, determined by body mass index (BMI) [9]. However, BMI has inherent limitations, as it relies on weight and height measurements [10, 11]. Consequently, BMI may not be a perfect indicator of obesity, particularly among men with higher muscle mass [12]. Furthermore, BMI fails to accurately assess the relationship between obesity and associated diseases due to its inability to account for variations in body fat distribution [13–15]. The commonly utilized pulmonary function parameters in the respiratory system include FVC, FEV1, and the FEV1/FVC ratio. The normal reference range for FVC is approximately 3000 ml to 5000 ml, while the normal reference range for FEV1 typically falls between 2000 ml and 4000ml [16]. However, these values are more influenced by factors such as age, gender, height, and weight [16]. A strong association between obesity, particularly abdominal obesity, and lung function has been established in the literature [17, 18].

In addition, obesity can be divided into android obesity (fat distribution in the chest, abdomen and internal organs) and gynoid obesity (fat distribution in the subcutaneous tissue of the limbs and buttocks) according to the characteristics of fat distribution [19]. This difference in fat distribution leads to android obesity having a more direct effect on lung mechanics than female obesity, because the increase in chest fat and the increase in abdominal volume can affect diaphragm contraction and reduce lung volume [20]. Not only that, android obesity will also secrete more pro-inflammatory adipokines because of its special fat distribution, aggravating the activation of immune cells and metabolic disorders [20].

However, existing research has focused mainly on children and adolescents, with mixed results. A study of Chinese people aged 20–80 years showed that WC was positively correlated with FEV1 and FVC [21] whereas another study of Chinese elderly people reported that an increase in WC was associated with a decrease in FEV1 and FVC [22]. Marga et al. [23] reported no significant association between WC and FVC or FEV1 in 8-year-olds. In contrast, Feng et al. [24] found that WC in Chinese children was negatively correlated with lung function. Zhang et al. [10] discovered that abdominal obesity was associated with impaired lung function among adults with asthma. Since the decline in lung function is closely related to changes in body size, we hypothesize that WC, independent of BMI, may be associated with impairment of lung function.

Therefore, our study aimed to use the National Health and Nutrition Survey (NHANES) database to investigate the correlation between WC and lung function in middle-aged and older adults. By using WC as a measure, we aim to elucidate the potential association between abdominal obesity and lung function in this particular population.

## Methods

### Study sample

**Fig. 1:**
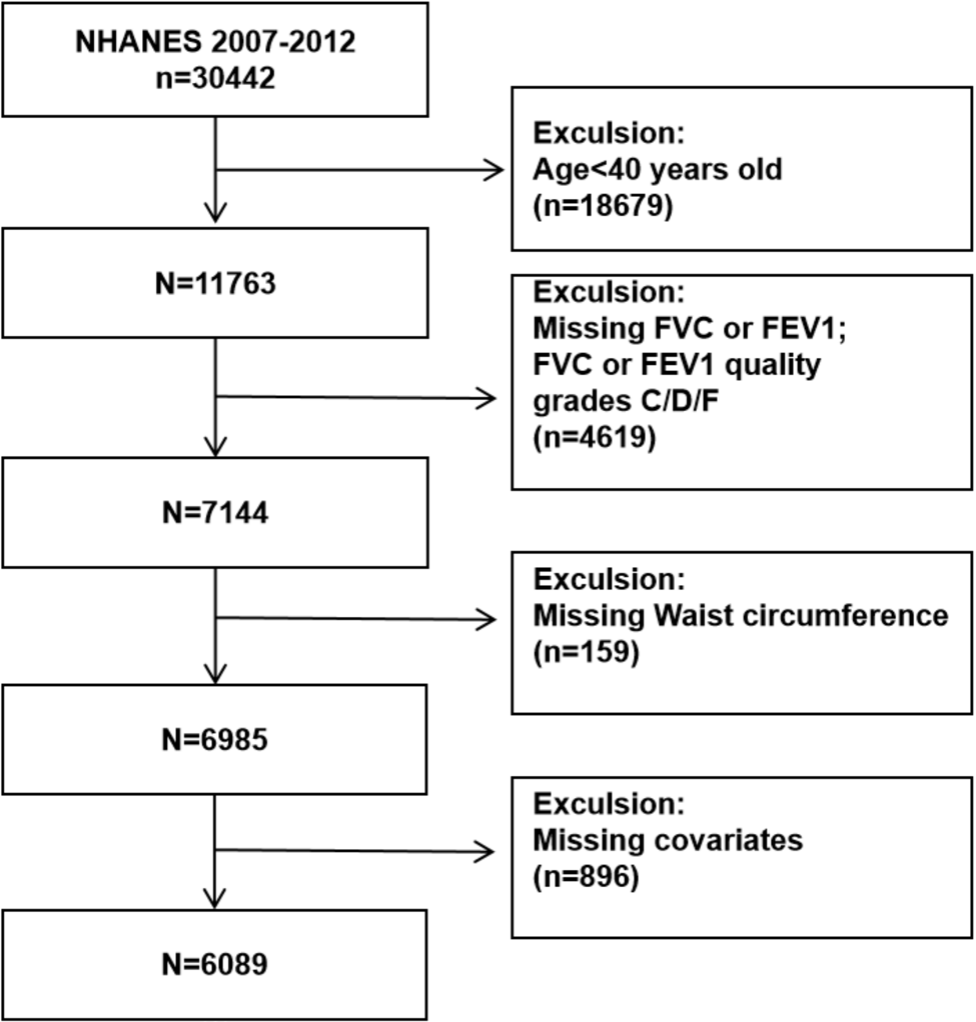
Flowchart for selecting analyzed participants FEV1, forced expiratory volume in one second; FVC, forced vital capacity; NHANES, National Health and Nutrition Examination Survey

The data for our study were sourced from the National Health and Nutrition Examination Survey (NHANES), a comprehensive survey conducted by the Centers for Disease Control and Prevention (CDC). Our study drew on data from NHANES spanning 2007 to 2012. The dataset comprises demographic, examination, laboratory, and questionnaire information. After an initial screening of the NHANES database, we identified that lung function data were available only for the period mentioned. Consequently, we included all participants (*n* = 30,442) from the NHANES conducted between 2007 and 2012. We excluded individuals (1) aged < 40 years old (*n* = 18,679) (2); missing lung function test results data (FEV1 or FVC) or having low data quality (C, D, F) (*n* = 4619) (3); missing WC data (*n* = 159) (4); missing data about covariates at least one of following (*n* = 896): BMI, the ratio of family income to poverty (PIR), total cholesterol, total bilirubin, total protein, aspartate aminotransferase (AST), or alanine aminotransferase (ALT). Ultimately, our study incorporated a substantial and nationally representative sample of middle-aged and older adults from the United States. A flowchart illustrating the screening process is presented in Fig. 1 for clarity. This study was approved by the ethics review board of the National Center for Health Statistics (NCHS) and obtained written informed consent from all participants.

### Lung function assessment

Lung function tests are performed by trained professional researchers and are tested in a standing position, unless the participant was physically limited. Lung function assessments were conducted using the Ohio 822/827 dry-roll volume spirometer, following the recommended guidelines from the American Thoracic Society (ATS) and the European Respiratory Society (ERS). The spirometry variables utilized in this study included FEV1, FVC, and the FEV1/FVC ratio. To ensure the reliability and accuracy of the spirometry measurements, the ATS/ERS criteria for acceptability and reproducibility were applied, resulting in spirometry quality grades ranging from A to F. Grades A and B indicated measurements that fulfilled or exceeded the ATS criteria. In contrast, grade C could still be considered for analysis. Grades D to F, conversely, were deemed less likely to be useful.

It is important to note that our study only included data with spirometry quality grades A and B for FEV1 and FVC. This rigorous selection criterion was employed to guarantee the accuracy and reliability of the measurement data while excluding data with lower quality grades (C, D, and F).

### Waist circumference measurement

WC measurements were conducted by trained health technicians in the Mobile Examination Center as part of the NHANES survey. The measurement procedure involved determining the waist circumference at the uppermost lateral border of the right ilium, with precision recorded to the nearest 0.1 cm.

### Other covariates

The criteria for selecting covariates in this study were: (1) demographic data; (2) variables affecting WC and lung function parameters in the published literature [25, 26]; (3) according to the recommendation of the STROBE statement, covariates with regression coefficients on the outcome variables with a P value < 0.10 or covariates that resulted in more than a 10% change in the regression coefficients of the risk factors after introduction of the covariates in the base model; (4) other variables accumulated on the basis of clinical experience.The demographic data consisted of age (in years), gender, race/ethnicity (including Mexican American, other Hispanic, non-Hispanic white, non-Hispanic black, and others), poverty-to-income ratio, educational level (categorized as less than high school, high school, and more than high school), and marital status (married, single, living with a partner). Furthermore, examination data and personal life history variables were included in our analysis. These variables encompassed BMI (in kg/m²), alcohol consumption (defined as having consumed at least 12 alcoholic drinks/1 year), smoking history (defined as having smoked at least 100 cigarettes in life), histories of diabetes, hypertension, and respiratory diseases. Last, laboratory data variables were incorporated, comprising measurements of total protein levels (in g/L), total cholesterol levels (in mmol/L), total bilirubin levels (in µmol/L), aspartate aminotransferase (AST) levels (in U/L), and alanine aminotransferase (ALT) levels (in U/L). For more detailed information regarding these variables, including specific measurement methods and ranges, (https://www.cdc.gov/nchs/nhanes/) provides comprehensive access to publicly available data.

### Statistical analysis

Statistical analyses were conducted following the guidelines provided by the Centers for Disease Control and Prevention (CDC) [CDC guideline criteria: https://wwwn.cdc.gov/nchs/nhanes/tutorials/default.aspx]. Continuous variables were reported as the means ± standard deviations (SD). Categorical variables are presented as percentages. Initially, weighted χ^2 tests were employed for categorical variables, while weighted linear regression models were used for continuous variables. Subsequently, we constructed four weighted linear regression models (Model 1, Model 2, Model 3, and Model 4), adjusting various variables to examine the association between WC and lung function parameters. A stratified analysis was also performed based on the fully adjusted model to explore potential stratified associations between WC and lung function. Additionally, a generalized additive model (GAM) with a penalty spline method was utilized to construct a smoothed curve-fitting fully adjusted model, treating WC as a continuous variable. We also calculated the variance inflation factor (VIF) for the variables, with VIF values of 5.8 and 6.6 for BMI and WC (supplementary Table 1), respectively. As a rule of thumb, the threshold for VIF values with multicollinearity between variables is 10 [27].

All statistical analyses were performed using Empower Stats software and R version 4.2.0. A p value of less than 0.05 was considered statistically significant in our study.

## Results

### Baseline characteristics of the participants

Table 1 shows the weighted distribution of baseline characteristics, including demographic, examination, laboratory, and questionnaire data, for the participants selected from the NHANES survey conducted between 2007 and 2012. A total of 6,089 participants aged 40–79 years were included in our study. The average age of the selected participants was 56.49 years (± 10.65), and non-Hispanic whites constituted the majority of the study population. The distribution of all included variables across the quartiles demonstrated statistically significant differences (p values < 0.05).

**Table 1:** Baseline characteristics of the selected participants

	Male	Female	*P* value
N	2983	3106	
Age, mean ± SD (years)	56.640 ± 10.686	56.344 ± 10.618	0.278
Race/ethnicity (%)			0.446
Mexican American	453 (15.186%)	441 (14.198%)	
Other Hispanic	296 (9.923%)	333 (10.721%)	
Non-Hispanic White	1420 (47.603%)	1490 (47.972%)	
Non-Hispanic Black	597 (20.013%)	641 (20.637%)	
Other Race	217 (7.275%)	201 (6.471%)	
Poverty to income ratio, mean ± SD	2.875 ± 1.659	2.716 ± 1.661	< 0.001
Education (%)			0.010
Less than high school	794 (26.617%)	764 (24.598%)	
High school	702 (23.533%)	676 (21.764%)	
More than high school	1487 (49.849%)	1663 (53.542%)	
Not recorded	0 (0.000%)	3 (0.097%)	
Marital status (%)			< 0.001
Married	2006 (67.248%)	1683 (54.185%)	
Single	798 (26.752%)	1309 (42.144%)	
Living with a partner	179 (6.001%)	114 (3.670%)	
BMI, mean ± SD	29.187 ± 5.566	29.871 ± 7.157	< 0.001
Had at least 12 alcohol drinks/1 year (%)			< 0.001
Yes	2434 (81.596%)	1816 (58.467%)	
No	411 (13.778%)	1129 (36.349%)	
Not recorded	138 (4.626%)	161 (5.184%)	
Smoked at least 100 cigarettes in life (%)			< 0.001
Yes	1726 (57.861%)	1268 (40.824%)	
No	1256 (42.105%)	1836 (59.111%)	
Not recorded	1 (0.034%)	2 (0.064%)	
Hypertension history(%)			0.994
Yes	1276 (42.776%)	1325 (42.659%)	
No	1704 (57.124%)	1778 (57.244%)	
Not recorded	3 (0.101%)	3 (0.097%)	
Diabetes history(%)			0.084
Yes	462 (15.488%)	431 (13.876%)	
No	2435 (81.629%)	2601 (83.741%)	
Borderline	86 (2.883%)	74 (2.382%)	
Respiratory diseases history(%)			< 0.001
Yes	547 (18.337%)	606 (19.511%)	
No	2380 (79.785%)	2358 (75.918%)	
Not recorded	56 (1.877%)	142 (4.572%)	
Total cholesterol, mean ± SD(mmol/L)	5.125 ± 1.104	5.367 ± 1.046	< 0.001
Total protein, mean ± SD (g/L)	71.700 ± 4.820	71.124 ± 4.638	< 0.001
Total bilirubin, mean ± SD (umol/L)	13.971 ± 5.141	11.474 ± 4.062	< 0.001
Alanine aminotransferase, mean ± SD (U/L)	29.696 ± 24.491	22.721 ± 14.800	< 0.001
Aspartate aminotransferase, mean ± SD (U/L)	28.677 ± 18.316	25.077 ± 13.604	< 0.001
FVC, mean ± SD (ml)	4319.430 ± 908.051	3030.188 ± 656.845	< 0.001
FEV1, mean ± SD (ml)	3225.306 ± 763.358	2336.456 ± 545.333	< 0.001
FEV1/FVC, mean ± SD (%)	74.608 ± 8.450	77.091 ± 7.317	< 0.001
Waist circumference (cm)	103.660 ± 14.419	98.598 ± 15.499	< 0.001

Mean ± SD for continuous variables: P value was calculated by a weighted linear regression model

% for categorical variables: P value was calculated by weighted chi-square test

### The associations between waist circumference and lung function parameters

Weighted multiple regression analysis was conducted to examine the association between WC and lung function parameters, as presented in Table 2. In males, both Model 1 and Model 2, representing unadjusted and age, race adjusted associations, revealed a negative correlation between WC and FVC as well as FEV1, while a positive correlation was observed with FEV1/FVC. In Model 3, which additionally adjusted for BMI based on Model 2, WC exhibited a positive correlation with FVC and FEV1, and a negative correlation with FEV1/FVC. Finally, in the fully adjusted Model 4, WC showed a positive correlation with FVC (β = 23.687, 95% CI: 18.523, 28.852) and FEV1 (β = 12.029, 95% CI: 7.789, 16.270), but a negative correlation with FEV1/FVC (β = -0.140, 95% CI: -0.192, -0.088).Similar results were observed in females and the total population. In fully adjusted analyses for females, WC exhibited a positive correlation with FVC (β = 6.583, 95% CI: 3.629, 9.538) and FEV1 (β = 4.453, 95% CI: 1.988, 6.918), and a negative correlation with FEV1/FVC (β = -0.034, 95% CI: -0.072, 0.004), although without statistical significance. In the fully adjusted analysis for the total population, WC showed a positive correlation with FVC (β = 12.014, 95% CI: 9.251, 14.777) and FEV1 (β = 6.557, 95% CI: 4.284, 8.831), and a negative correlation with FEV1/FVC (β = -0.076, 95% CI: -0.107, -0.046). Due to partial collinearity between WC and BMI, we assessed individual associations between WC, BMI, and pulmonary function to elucidate the potential mediating role of BMI in the relationship between WC and pulmonary function (Supplementary Figs. 1–4).

**Table 2:** Multivariate weighted regression model analysis reveals the associations between waist circumference and lung function parameters

	Model 1	Model 2	Model 3	Model 4
	β (95% CI) *P* value	β (95% CI) *P* value	β (95% CI) *P* value	β (95% CI) *P* value
Male				
FVC	-8.931 (-11.218, -6.645) < 0.001	-8.463 (-10.434, -6.492) < 0.001	19.746 (14.468, 25.024) < 0.001	23.687 (18.523, 28.852) < 0.001
FEV1	-6.048 (-7.980, -4.117) < 0.001	-4.241 (-5.851, -2.632) < 0.001	7.119 (2.738, 11.500) < 0.001	12.029 (7.789, 16.270) < 0.001
FEV1/FVC	0.005 (-0.016, 0.026) 0.646	0.038 (0.019, 0.058) 0.001	-0.187 (-0.240, -0.133) < 0.001	-0.140 (-0.192, -0.088) < 0.001
Female				
FVC	-9.894 (-11.352, -8.435) < 0.001	-7.147 (-8.347, -5.947) < 0.001	3.451 (0.475, 6.426) 0.023	6.583 (3.629, 9.538) 0.001
FEV1	-5.465 (-6.698, -4.233) < 0.001	-3.090 (-4.090, -2.090) < 0.001	1.154 (-1.344, 3.652) 0.365	4.453 (1.988, 6.918) 0.004
FEV1/FVC	0.068 (0.051, 0.084) < 0.001	0.077 (0.062, 0.093) < 0.001	-0.064 (-0.102, -0.025) 0.001	-0.034 (-0.072, 0.004) 0.079
Total				
FVC	-9.489 (-10.795, -8.182) < 0.001	-7.385 (-8.493, -6.276) < 0.001	8.638 (5.839, 11.437) < 0.00001	12.014 (9.251, 14.777) < 0.001
FEV1	-5.711 (-6.814, -4.607) < 0.001	-3.361 (-4.273, -2.448) < 0.001	2.697 (0.369, 5.024) 0.02319	6.557 (4.284, 8.831) < 0.001
FEV1FVC	0.041 (0.028, 0.054) < 0.001	0.062 (0.049, 0.074) < 0.001	-0.113 (-0.145, -0.082) < 0.00001	-0.076 (-0.107, -0.046) < 0.001

Model 1: no covariates were adjusted

Model 2: age and race/ethnicity were adjusted

Model 3: Model 2 plus body mass index was adjusted

Model 4: Model 3 plus education, poverty-to-income ratio, marital status, at least 12 alcohol drinks/1 year, smoking at least 100 cigarettes in life, hypertension history, diabetes history, respiratory disease history, total cholesterol, total protein, total bilirubin, alanine aminotransferase, and aspartate aminotransferase were adjusted

### Stratified associations between waist circumference and lung function parameters

To assess the stability of the multivariate regression analysis results, we conducted stratified analyses to examine the associations between WC and lung function parameters in different subgroups. The results are presented in Table 3.

**Table 3:** The stratified analysis of the association between waist circumference and lung function parameters

	*N*	FVC	FEV1	FEV1/FVC
		β (95% CI) *P* value	β (95% CI) *P* value	β (95% CI) *P* value
Age				
40–60	3849	8.615 (5.011, 12.218) < 0.0001	3.452 (0.392, 6.512) 0.0271	-0.084 (-0.121, -0.047) < 0.0001
>60	2240	7.464 (2.790, 12.138) 0.0018	1.607 (-2.134, 5.349) 0.3999	-0.126 (-0.180, -0.071) < 0.0001
P for interaction		0.9820	0.7510	0.2897
Gender				
Male	2983	23.687 (18.523, 28.852) < 0.0001	12.029 (7.789, 16.270) < 0.0001	-0.140 (-0.192, -0.088) < 0.0001
Female	3106	6.583 (3.629, 9.538) < 0.0001	4.453 (1.988, 6.918) 0.0004	-0.034 (-0.072, 0.004) 0.0797
P for interaction		< 0.0001	0.0002	0.0295
Race				
Mexican American	894	16.932 (9.631, 24.233) < 0.0001	10.167 (4.468, 15.866) 0.0005	-0.100 (-0.170, -0.030) 0.0049
Other Hispanic	629	13.779 (5.116, 22.441) 0.0019	8.138 (1.199, 15.076) 0.0219	-0.101 (-0.199, -0.004) 0.0415
Non-Hispanic White	2910	11.524 (7.434, 15.614) < 0.0001	6.009 (2.634, 9.385) 0.0005	-0.081 (-0.126, -0.035) 0.0005
Non-Hispanic Black	1238	8.934 (4.031, 13.838) 0.0004	6.369 (2.182, 10.557) 0.0029	-0.026 (-0.088, 0.035) 0.4040
Other Race	418	6.456 (-5.089, 18.001) 0.2737	4.183 (-5.022, 13.387) 0.3737	-0.011 (-0.131, 0.109) 0.8631
P for interaction		0.2658	0.3522	0.5110
Education				
Less than high school	1558	3.449 (-2.139, 9.038) 0.2266	-0.873 (-5.496, 3.750) 0.7113	-0.121 (-0.190, -0.053) 0.0005
High school	1378	11.357 (5.544, 17.170) 0.0001	3.145 (-1.716, 8.005) 0.2050	-0.146 (-0.215, -0.078) < 0.0001
More than high school	3150	14.693 (10.855, 18.531) < 0.0001	9.844 (6.718, 12.970) < 0.0001	-0.038 (-0.078, 0.001) 0.0586
P for interaction		0.4411	0.0111	0.0444
Marital status				
Married	3689	12.692 (9.021, 16.364) < 0.0001	7.692 (4.725, 10.659) < 0.0001	-0.060 (-0.099, -0.021) 0.0027
Single	2107	10.992 (6.756, 15.229) < 0.0001	4.964 (1.335, 8.593) 0.0074	-0.099 (-0.149, -0.049) 0.0001
Living with a partner	293	5.333 (-10.584, 21.249) 0.5120	-2.083 (-15.075, 10.909) 0.7536	-0.140 (-0.327, 0.047) 0.1448
P for interaction		0.0185	0.0099	0.7537
Poverty to income ratio				
Low	1999	12.459 (7.701, 17.217) < 0.0001	8.013 (4.065, 11.960) < 0.0001	-0.066 (-0.122, -0.010) 0.0207
Middle	2057	11.671 (6.806, 16.536) < 0.0001	5.078 (1.062, 9.094) 0.0133	-0.114 (-0.169, -0.060) < 0.0001
High	2033	12.244 (7.503, 16.985) < 0.0001	7.095 (3.207, 10.984) 0.0004	-0.054 (-0.104, -0.003) 0.0388
P for interaction		0.0582	0.0254	0.3353
BMI				
<=25	1465	13.053 (8.146, 17.959) < 0.0001	7.570 (3.485, 11.654) 0.0003	-0.047 (-0.106, 0.011) 0.1107
25–30	2185	6.109 (1.583, 10.635) 0.0082	3.072 (-0.575, 6.720) 0.0989	-0.026 (-0.075, 0.022) 0.2910
> 30	2439	-1.931 (-4.135, 0.272) 0.0860	-2.133 (-3.956, -0.310) 0.0219	-0.012 (-0.036, 0.011) 0.2974
P for interaction		< 0.0001	< 0.0001	0.3046
Smoked at least 100 cigarettes in life				
Yes	2994	11.871 (7.642, 16.100) < 0.0001	3.928 (0.313, 7.542) 0.0333	-0.138 (-0.189, -0.087) < 0.0001
No	3092	12.849 (9.209, 16.490) < 0.0001	9.181 (6.342, 12.019) < 0.0001	-0.023 (-0.058, 0.012) 0.1891
P for interaction		0.0038	0.0056	0.9716
Had at least 12 alcohol drinks/1 year				
Yes	4250	13.725 (10.269, 17.181) < 0.0001	7.693 (4.856, 10.530) < 0.0001	-0.080 (-0.117, -0.042) < 0.0001
No	1540	7.873 (3.209, 12.538) 0.0010	3.499 (-0.405, 7.402) 0.0792	-0.073 (-0.128, -0.017) 0.0107
P for interaction		0.0276	0.0203	0.1413
Diabetes history				
Yes	893	7.164 (0.865, 13.463) 0.0261	1.831 (-3.582, 7.243) 0.5076	-0.080 (-0.157, -0.002) 0.0434
No	5036	13.354 (10.245, 16.463) < 0.0001	7.502 (4.955, 10.049) < 0.0001	-0.079 (-0.113, -0.045) < 0.0001
Borderline	160	-2.285 (-19.305, 14.736) 0.7929	-1.533 (-15.620, 12.554) 0.8314	0.029 (-0.141, 0.200) 0.7379
P for interaction		0.0001	< 0.0001	0.5750
Respiratory diseases history				
Yes	1153	6.815 (0.565, 13.065) 0.0328	2.550 (-2.775, 7.874) 0.3481	-0.086 (-0.160, -0.013) 0.0215
No	4738	13.614 (10.434, 16.794) < 0.0001	7.637 (5.038, 10.236) < 0.0001	-0.078 (-0.112, -0.044) < 0.0001
P for interaction		0.7381	0.4648	0.6864
Hypertension history				
Yes	2601	11.331 (7.364, 15.299) < 0.0001	8.076 (4.732, 11.420) < 0.0001	-0.028 (-0.075, 0.018) 0.2305
No	3482	13.148 (9.267, 17.029) < 0.0001	6.018 (2.877, 9.160) 0.0002	-0.105 (-0.146, -0.063) < 0.0001
P for interaction		0.7472	0.2766	0.0706

The above adjusted for gender, age, race, education, marital status, PIR, BMI, at least 12 alcohol drinks/1 year, smoking at least 100 cigarettes in life, hypertension history, diabetes history, respiratory disease history, total cholesterol, total protein, total bilirubin, alanine aminotransferase, and aspartate aminotransferase. In each case, the model was not adjusted for the stratification variable itself

In the subgroup analyses, WC demonstrated a positive relationship with FVC in most subgroups, except for the subgroup of other races, less than high school, living with a partner, BMI > 30, and borderline diabetes history. Similarly, WC showed a positive relationship with FEV1 in most subgroups, except for the subgroup of age > 60, other race, less than high school, high school, living with a partner, BMI25-30, BMI > 30 (negative correlation with statistical significance), at least 12 alcohol drinks/1 year, with diabetes history, borderline diabetes history, and respiratory diseases history. On the other hand, WC exhibited a negative relationship with FEV1/FVC in most subgroups, except for the non-Hispanic Black, other race, more than high school, living with a partner, all BMI subgroups, no smoking, borderline diabetes history and hypertension history subgroups. Furthermore, gender and BMI have a significant interaction with FVC (p for interaction < 0.0001); BMI and diabetes history have a significant interaction with FEV1 (p for interaction < 0.0001).

### Using GAM to explore the possible relationship between waist circumference and lung function parameters

**Fig. 2:**
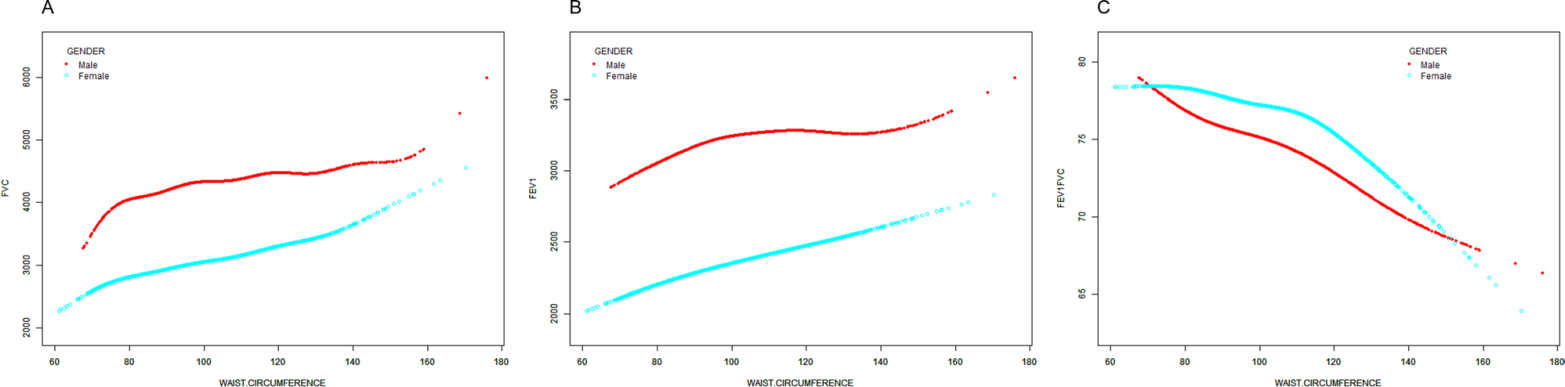
Based on the fully adjusted model, the relationship between waist circumference and lung function

To ensure the reliability of the regression analysis results, we used a generalized additive model (GAM) to investigate whether there is a linear or nonlinear correlation between WC and lung function parameters. In our study, based on Model 4 (adjusted for all covariates), we constructed a smooth-fitting curve to observe potential correlations. Figure 2 shows the results obtained from the GAM analysis. We observed a nonlinear relationship between WC and lung function parameters. After adjusting for all covariates, we found that WC, FVC and FEV1 were positively correlated and nonlinear. Conversely, we observe a nonlinear negative correlation between WC and FEV1/FVC ratios. With the increase of WC, the FEV1/FVC ratio tends to decrease.

## Discussion

To our knowledge, there has been limited investigation into the relationship between WC and lung function parameters in middle-aged and older adults in the United States, accounting for the influence of BMI. We investigated the correlation between WC and lung function parameters in 6089 middle-aged and older adults who participated in the NHANES survey in the United States between 2007 and 2012. Four weighted multiple linear regression models were used to determine the relationship between WC and three lung function parameters. Based on NHANES data from 2007 to 2012, we found that WC was negatively associated with FVC and FEV1 and positively associated with FEV1/FVC in the unadjusted model and after adjusting for age and race. After adjusting for BMI, the correlation between WC and FVC and FEV1 became positive, and the correlation with FEV1/FVC became negative. Finally, the correlation between WC and lung function parameters in the fully adjusted model was the same as above (Male: FVC, β = 23.687; FEV1, β = 12.029; FEV1/FVC, β = -0.140; Female: FVC, β = 6.583; FEV1, β = 4.453; FEV1/FVC, β = -0.034; Total population: FVC, β = 12.014; FEV1, β = 6.557; FEV1/FVC, β=-0.076). To verify the accuracy and stability of this association, we performed a stratified analysis. Then, we build a smooth curve model to further assess the reliability of the results.

Our study results indicate an association between increased WC and decreased FEV1/FVC ratio, aligning with the majority of previously published findings. A study by Zhang et al. [28]. in American adults found that abdominal obesity was associated with an increased risk of airflow obstruction defined by FEV1/FVC. A cohort study in the Netherlands by Marga et al. [23]. found that large WC in girls only, independent of BMI, was associated with lower FEV1/FVC. Feng et al. [24]. found that waist-to-chest ratio (WCR) was negatively correlated with FVC, FEV1, FVC/FEV1 in Chinese adolescents and children, after adjusting for gender height and BMI. Chen et al [29]. found that an increase in WC in children aged 6–17 years is associated with an increase in FVC and FEV1, while it is associated with a decrease in the FEV1/FVC ratio. With respect to FVC and FEV1, Zeng et al. [21]. discovered that in the Chinese population aged 20–80 years, WC and obesity defined by WC are positively correlated with FVC and FEV1. A cohort study by Pan et al. [22]. reported that abdominal obesity and its indicators (WC, WHtR, WHR and body fat) were associated with decreased FVC and FEV1 in the older Chinese population. Zhang et al. [10] reported that in adult asthma patients in the United States, the abdominal obesity group was associated with lower FVC and FEV1 compared to the normal group. Our data reveals that in the model without adjusting for BMI, WC is negatively correlated with FVC and FEV1, while after adjusting for BMI, it exhibits a positive correlation. These divergent conclusions about FVC and FEV1 may be attributed to differences in study designs, study population, and the confounding factors included, particularly BMI.

Central obesity is a specific type of obesity characterized by the accumulation of fat in the chest, abdomen, and internal organs [30]. Obesity reduces respiratory compliance, alters breathing patterns, affecting lung function [19, 31]. The fatty deposition also causes narrowing, closure, and hyperresponsiveness of the airways, resulting in uneven ventilation [32, 33]. Excess body fat alters respiratory physiology and impairs lung function [34]. Abdominal fat accumulation can affect the contraction of the diaphragm and impair lung function. The effect of intra-abdominal pressure on the diaphragm is one of the important reasons for the impairment of lung function [14, 35]. Thus, abdominal obesity leads to decreased lung compliance, increased airway resistance, and limited daily exercise [19, 36]. People with abdominal obesity may also change their breathing pattern to rapid and shallow breathing. This style of breathing increases the risk of airflow limitation, hypoxia, respiratory overload, and respiratory complications [37]. In addition, inflammation and oxidative stress have been identified as key factors in impaired lung function due to abdominal obesity [38, 39]. Systemic adipose tissue inflammation may be responsible for impaired lung function due to abdominal obesity [40]. Abdominal obesity is considered to be an inflammatory state [18], and many inflammatory factors come from visceral adipose tissue, such as IL-6, TNF-α, C-reactive protein (CRP), leptin, etc., which may lead to obesity-related airway inflammation [41]. In addition, CRP is also thought to cause impairment of lung function [42]. An in vitro study found that CRP is present in human respiratory secretions [43] and may play a local role in lung tissue, decreasing airway diameter and lung function [18, 44]. Besides, studies have demonstrated that the relationship between lung function and abdominal obesity is also affected by CRP gene polymorphisms. The researchers found that the CRP rs1205 CC genotype was associated with impaired lung function [45], suggesting that the CRP gene plays a partial role in lung function inheritance.

### Study strengths and limitations

Compared with previously published articles, our study has the following advantages. First, our sample includes 6089 nationally representative middle-aged and older adults, and the sample size is relatively large. Second, we have taken into account BMI, an important confounding factor, so that WC as an indicator of abdominal fat deposits can be understood in the context of body type so that we can understand its full impact on respiratory function. Also, we performed a stratified analysis that considered the possible impact of BMI and other confounding factors on the results, which helped verify the reliability of the results and identify possible susceptible populations. Finally, based on completely adjusting the model, we performed smooth curve fitting and explored the relationship between WC and lung function parameters.

However, it should be noted that our study design is a cross-sectional study and cannot prove a causal relationship between abdominal obesity and altered lung function, so more prospective cohort studies are needed to validate the conclusions. Second, we chose WC as a marker of abdominal obesity, while other markers, such as waist-to-height ratio or waist-hip ratio, were not included in the study due to lack of data or small sample sizes. Future studies are needed to confirm our results with other methods of measuring abdominal obesity. Third, while we adjusted for many confounders, other potential confounding factors were not considered, similar to other cross-sectional studies. Finally, our survey is based on the NHANES database, which applies to the US population and, therefore, is geographically limited in versatility. More comprehensive studies are needed to determine the relationship between WC and lung function parameters.

## Conclusion

Our findings indicate that in the fully adjusted model, waist circumference, independent of BMI, positively correlates with FVC and FEV1 while exhibiting a negative correlation with FEV1/FVC among middle-aged and older adults in the United States. These results underscore the importance of considering abdominal obesity as a potential factor influencing lung function in American middle-aged and older adults.

### Electronic supplementary material

Below is the link to the electronic supplementary material.

Supplementary Material 1

## Data Availability

No datasets were generated or analysed during the current study.

## References

[1] 2015 GBD, Collaborators O, Afshin A, Forouzanfar MH, Reitsma MB, Sur P, Estep K et al. Health Effects of Overweight and Obesity in 195 Countries over 25 Years. N Engl J Med. 2017;377:13–27. 10.1056/NEJMoa1614362 PMC5477817 28604169

[2] Chan M (2017). Obesity and diabetes: the slow-motion disaster. Milbank Q.

[3] Chooi YC, Ding C, Magkos F (2019). The epidemiology of obesity. Metabolism.

[4] Kelly T, Yang W, Chen C-S, Reynolds K, He J. Global burden of obesity in 2005 and projections to 2030. Int J Obes. 2005. 2008;32:1431–7. 10.1038/ijo.2008.102 18607383

[5] McClean KM, Kee F, Young IS, Elborn JS (2008). Obesity and the lung: 1. Epidemiology. Thorax.

[6] Avgerinos KI, Spyrou N, Mantzoros CS, Dalamaga M (2019). Obesity and cancer risk: emerging biological mechanisms and perspectives. Metabolism.

[7] Melo LC, Silva MAM da, Calles AC do N. Obesity and lung function: a systematic review. Einstein Sao Paulo Braz. 2014;12:120–5. 10.1590/S1679-45082014RW2691 PMC4898251 24728258

[8] Matrone A, Ferrari F, Santini F, Elisei R. Obesity as a risk factor for thyroid cancer. Curr Opin Endocrinol Diabetes Obes. 2020;27:358–63. 10.1097/MED.0000000000000556 32740043

[9] Ross R, Neeland IJ, Yamashita S, Shai I, Seidell J, Magni P, et al. Waist circumference as a vital sign in clinical practice: a Consensus Statement from the IAS and ICCR Working Group on visceral obesity. Nat Rev Endocrinol. 2020;16:177–89. 10.1038/s41574-019-0310-7 PMC7027970 32020062

[10] Zhang H, Hu Z, Wang S, Xu J, Li S, Song X (2023). Association of general and abdominal obesity with lung function, FeNO, and blood eosinophils in adult asthmatics: findings from NHANES 2007–2012. Front Physiol.

[11] Bracht JR, Vieira-Potter VJ, De Souza Santos R, Öz OK, Palmer BF, Clegg DJ (2020). The role of estrogens in the adipose tissue milieu. Ann N Y Acad Sci.

[12] von Hafe P, Pina F, Pérez A, Tavares M, Barros H (2004). Visceral fat accumulation as a risk factor for prostate cancer. Obes Res.

[13] Piché M-E, Poirier P, Lemieux I, Després J-P (2018). Overview of epidemiology and contribution of obesity and body Fat distribution to Cardiovascular Disease: an update. Prog Cardiovasc Dis.

[14] Després J-P, Lemieux I (2006). Abdominal obesity and metabolic syndrome. Nature.

[15] Wehrmeister FC, Menezes AMB, Muniz LC, Martínez-Mesa J, Domingues MR, Horta BL (2012). Waist circumference and pulmonary function: a systematic review and meta-analysis. Syst Rev.

[16] Quanjer PH, Stanojevic S, Cole TJ, Baur X, Hall GL, Culver BH (2012). Multi-ethnic reference values for spirometry for the 3–95-yr age range: the global lung function 2012 equations. Eur Respir J.

[17] Leone N, Courbon D, Thomas F, Bean K, Jégo B, Leynaert B (2009). Lung function impairment and metabolic syndrome: the critical role of abdominal obesity. Am J Respir Crit Care Med.

[18] He H, Wang B, Zhou M, Cao L, Qiu W, Mu G (2020). Systemic inflammation mediates the associations between abdominal obesity indices and lung function decline in a Chinese General Population. Diabetes Metab Syndr Obes Targets Ther.

[19] Dixon AE, Peters U (2018). The effect of obesity on lung function. Expert Rev Respir Med.

[20] Palma G, Sorice GP, Genchi VA, Giordano F, Caccioppoli C, D’Oria R (2022). Adipose tissue inflammation and pulmonary dysfunction in obesity. Int J Mol Sci.

[21] Zeng X, Liu D, An Z, Li H, Song J, Wu W (2021). Obesity parameters in relation to lung function levels in a large Chinese rural adult population. Epidemiol Health.

[22] Pan J, Xu L, Lam TH, Jiang CQ, Zhang WS, Jin YL (2017). Association of adiposity with pulmonary function in older Chinese: Guangzhou Biobank Cohort Study. Respir Med.

[23] Bekkers MBM, Wijga AH, de Jongste JC, Kerkhof M, Postma D, Gehring U (2013). Waist circumference, BMI, and lung function in 8-year-old children: the PIAMA birth cohort study. Pediatr Pulmonol.

[24] Feng K, Chen L, Han S-M, Zhu G-J (2012). Ratio of waist circumference to chest circumference is inversely associated with lung function in Chinese children and adolescents. Respirol Carlton Vic.

[25] Wen J, Wei C, Giri M, Zhuang R, Shuliang G (2023). Association between serum uric acid/serum creatinine ratios and lung function in the general American population: National Health and Nutrition Examination Survey (NHANES), 2007–2012. BMJ Open Respir Res.

[26] Chen J, Zhu L, Yao X, Zhu Z (2023). The association between abdominal obesity and femoral neck bone mineral density in older adults. J Orthop Surg.

[27] Everitt BS, Howell DC (2005). Encyclopedia of statistics in behavioral science.

[28] Zhang X, Chen H, Gu K, Jiang X (2022). Association of Body Mass Index and Abdominal obesity with the risk of airflow obstruction: National Health and Nutrition Examination Survey (NHANES) 2007–2012. COPD J Chronic Obstr Pulm Dis.

[29] Chen Y, Rennie D, Cormier Y, Dosman JA (2009). Waist circumference associated with pulmonary function in children. Pediatr Pulmonol.

[30] Molani Gol R, Rafraf M (2021). Association between abdominal obesity and pulmonary function in apparently healthy adults: a systematic review. Obes Res Clin Pract.

[31] Littleton SW (2012). Impact of obesity on respiratory function. Respirol Carlton Vic.

[32] Chapman DG, Berend N, King GG, Salome CM (2008). Increased airway closure is a determinant of airway hyperresponsiveness. Eur Respir J.

[33] Littleton SW, Tulaimat A (2017). The effects of obesity on lung volumes and oxygenation. Respir Med.

[34] Agudelo CW, Samaha G, Garcia-Arcos I (2020). Alveolar lipids in pulmonary disease. A review. Lipids Health Dis.

[35] Grassi L, Kacmarek R, Berra L (2020). Ventilatory mechanics in the patient with obesity. Anesthesiology.

[36] Chen Y, Rennie D, Cormier YF, Dosman J (2007). Waist circumference is associated with pulmonary function in normal-weight, overweight, and obese subjects. Am J Clin Nutr.

[37] Kwon H, Kim D, Kim JS (2017). Body Fat distribution and the risk of Incident Metabolic Syndrome: a longitudinal cohort study. Sci Rep.

[38] Mu G, Zhou Y, Ma J, Guo Y, Xiao L, Zhou M (2019). Combined effect of central obesity and urinary PAH metabolites on lung function: a cross-sectional study in urban adults. Respir Med.

[39] Arteaga-Solis E, Zee T, Emala CW, Vinson C, Wess J, Karsenty G (2013). Inhibition of leptin regulation of parasympathetic signaling as a cause of extreme body weight-associated asthma. Cell Metab.

[40] Saltiel AR, Olefsky JM (2017). Inflammatory mechanisms linking obesity and metabolic disease. J Clin Invest.

[41] Mancuso P (2010). Obesity and lung inflammation. J Appl Physiol Bethesda Md 1985.

[42] Ren Z, Zhao A, Wang Y, Meng L, Szeto IM-Y, Li T (2018). Association between Dietary Inflammatory Index, C-Reactive protein and metabolic syndrome: a cross-sectional study. Nutrients.

[43] Gould JM, Weiser JN (2001). Expression of C-reactive protein in the human respiratory tract. Infect Immun.

[44] Määttä AM, Kotaniemi-Syrjänen A, Malmström K, Malmberg LP, Sundvall J, Pelkonen AS (2017). Vitamin D, high-sensitivity C-reactive protein, and airway hyperresponsiveness in infants with recurrent respiratory symptoms. Ann Allergy Asthma Immunol off Publ Am Coll Allergy Asthma Immunol.

[45] Sunyer J, Pistelli R, Plana E, Andreani M, Baldari F, Kolz M (2008). Systemic inflammation, genetic susceptibility and lung function. Eur Respir J.

