# Supplementary material for: Association between waist circumference and lung function in American middle-aged and older adults: findings from NHANES 2007–2012
Source: J Health Popul Nutr. 2024 Jun 26;43:98. doi: 10.1186/s41043-024-00592-6 (PMC11209998; doi:10.1186/s41043-024-00592-6)
Supplement: Supplementary file 1 — Supplementary Material 1 [file 41043_2024_592_MOESM1_ESM.docx]

| Variables | VIF |
| --- | --- |
| Age | 1.2 |
| Gender | 1.5 |
| Race | 1.1 |
| Education | 1.3 |
| Marital status | 1.1 |
| PIR | 1.3 |
| TC | 1.1 |
| BMI | 5.8 |
| Smoked at least 100 cigarettes in life | 1.1 |
| Diabetes history | 1.1 |
| WC | 6.6 |
| Had at least 12 alcohol drinks/1 year | 1.1 |
| ALT | 3.4 |
| AST | 3.3 |
| TB | 1.2 |
| Respiratory diseases history | 1 |
| TP | 1.1 |
| Hypertension history | 1.2 |

VIF: variance inflation factors. VIF for a variable = 1/(1-R²), where R² is the R-squared of the regression model of that variable against all other variables (e.g. X1=X2+X3+...)

**Supplement Table 1 Variables collinearity VIF value**

**
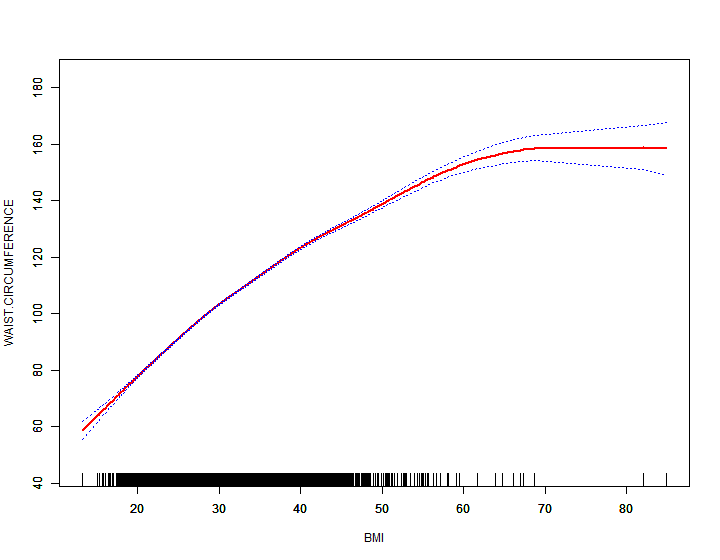
 Supplement Fig. 1 Association between body mass index and waist circumference.**


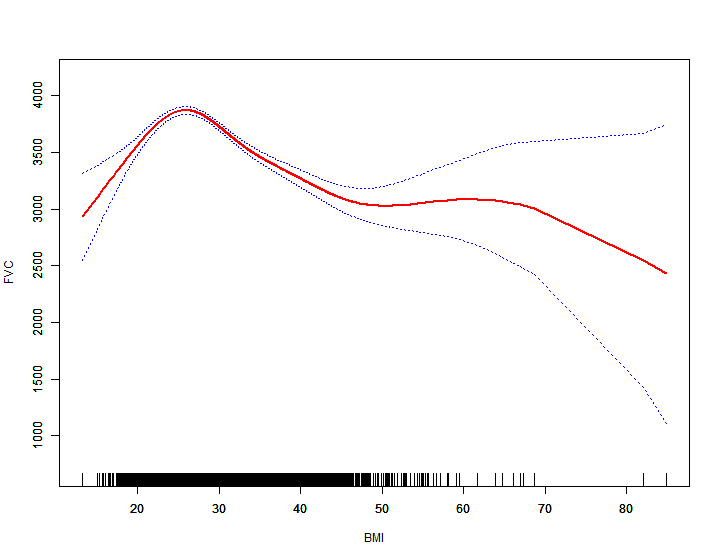
 **Supplement Fig. 2 Association between body mass index and FVC.**


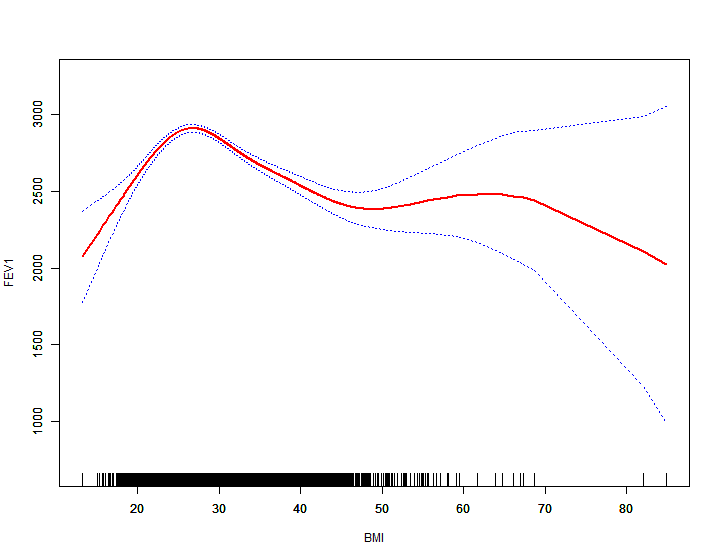
 **Supplement Fig. 3 Association between body mass index and FEV1.**


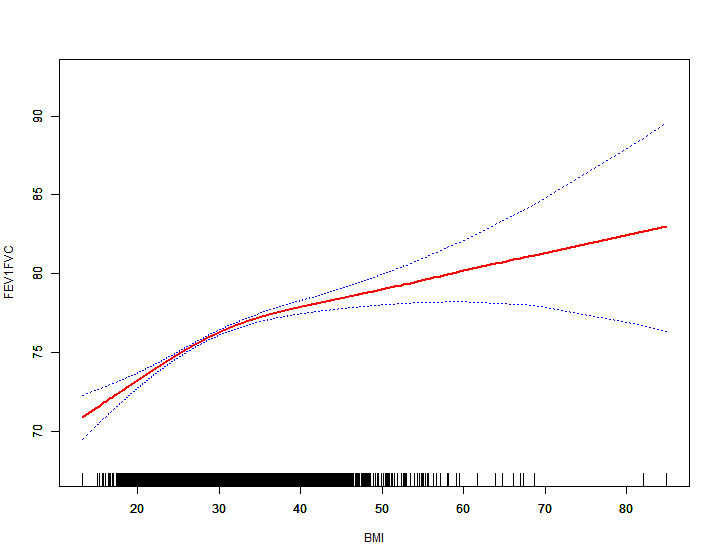
 **Supplement Fig. 4 Association between body mass index and FEV1/FVC.**
